# Long-menu questions in computer-based assessments: a retrospective observational study

**DOI:** 10.1186/s12909-016-0578-4

**Published:** 2016-02-09

**Authors:** Bernard Cerutti, Katherine Blondon, Annick Galetto

**Affiliations:** Unit of Research and Development in Medical Education, Faculty of Medicine, University Geneva, 1 Rue Michel Servet, Geneva, 1211 Switzerland; Division of General Internal Medicine, Geneva University Hospitals, Geneva, Switzerland; Division of Paediatric Emergency Medicine, Department of Child and Adolescent Medicine, Geneva University Hospitals, Geneva, Switzerland

**Keywords:** Long-menu questions, Computer-based assessment, Exam

## Abstract

**Background:**

Computer based assessments of paediatrics in our institution use series of clinical cases, where information is progressively delivered to the students in a sequential order. Three types of formats are mainly used: Type A (single answer), Pick N, and Long-menu. Long-menu questions require a long, hidden list of possible answers: based on the student’s initial free text response, the program narrows the list, allowing the student to select the answer. This study analyses the psychometric properties of Long-menu questions compared with the two other commonly used formats: Type A and Pick N.

**Methods:**

We reviewed the difficulty level and discrimination index of the items in the paediatric exams from 2009 to 2015, and compared the Long-menu questions with the Type A and Pick N questions, using multiple-way analyses of variances.

**Results:**

Our dataset included 13 exam sessions with 855 students and 558 items included in the analysis, 212 (38 %) Long-menu, 201 (36 %) Pick N, and 140 Type A (25 %) items. There was a significant format effect associated with both level of difficulty (*p* = .005) and discrimination index (*p* < .001). Long-menu questions were easier than Type A questions(+5.2 %; 95 % CI 1.1–9.4 %), and more discriminative than both Type A (+0.07; 95 % CI 0.01–0.14), and Pick N (+0.10; 95 % CI 0.05–0.16) questions.

**Conclusions:**

Long-menu questions show good psychometric properties when compared with more common formats such as Type A or Pick N, though confirmatory studies are needed. They provide more variety, reduce the cueing effect, and thus may more closely reflect real life practice than the other item formats inherited from paper-based examination that are used during computer-based assessments.

## Background

The use of computer-based assessments (CBA) has increased dramatically over the last decade [1]. This CBA adoption growth arises from the decreasing cost of information technology equipment, and the development and availability of specific hardware and software solution. CBA allows the use of many different kinds of media, facilitates prompt and more timely feedback to both students and teachers [1], and enhances of the acceptance of formative exams [2], without affecting performance in comparison with paper-based assessments [3]. Furthermore, students’ perceptions of advantages of CBA outweigh any disadvantages [4]. However, paper-based exam with computer support for the analyses of scanned answer sheets remains the most cost-effective solution, according to Mandel et al. [5], and CBA only pays off when new types of tasks are used.

CBA innovations include a new question format called “Long-menu questions”, which bear many positive features. This format assesses decision-making during diagnostic evaluation, diagnosis, and therapy [6]: the program narrows down the potential answers while the students type in their free text response, leaving the student with a number of options for their final selection. For that reason, Long-menu questions cannot be used in paper-based exams. The hidden list of potential answers may be extremely long. For example, the whole international classification of diseases [7] can be used for a question on diagnoses. This new format bears several advantages: potential time gain, straightforward scoring and reduced cuing effect. Correcting and scoring of Long-menu questions is rapid, compared with short-answer questions which require manual corrections by one or several examiners. Cuing effect and sheer guessing are decreased [8], as students must start typing their answer before having options to choose from, rather than simply choosing from a given list. Furthermore, response time for both Long-menu and open-ended questions are longer than for multiple choice questions. Finally, Long-menu questions show no difference in level of difficulty compared with short answer, open-ended questions or multiple choice questions [9]. Despite all these potential advantages, it is not known whether long-menu questions perform better than other question formats used in written assessments.

According to Dillon et al. [10], computer-based case simulations can be viewed as a simulation that falls between multiple choice questions, which provide an assessment of the proficiency in applying knowledge to descriptions of a clinical situation, and exams with standardized patients, which provide a realistic context for measuring the skills involved in taking history and performing physical examination. This assertion is corroborated by the situation in our institution: the CBA of paediatrics if one of the best predictive intra mural exams regarding the performance of our students during the national licensing examination (unpublished data). This licensing examination takes place at the end of the 6-year medical curriculum, and includes both a multiple choice question and a standardized patients components.

The main objective of this study was to measure the level of difficulty and power of discrimination of the Long-menu questions in the paediatric CBA, and to compare them with the other common assessment formats (Type A and Pick N).

## Methods

This retrospective study included of all the questions of the paediatric CBA from 2009 (introduction of CBA) to May 2015**.**

### Training in paediatrics

The training course in paediatrics in our institution is integrated in the fourth year (in a 6-year program) of the medical curriculum. The paediatrics rotation has a duration of 8 weeks, and runs concurrently with rotations in internal medicine, family medicine, surgery and a combination of psychiatry, gynaecology and obstetrics.

### Summative assessment

The exams take place twice a year: a mid-year session occurs once two groups of students have completed their paediatrics rotation, and a second session, for the remaining three groups of students of the academic year, occurs at the end of the year. Students are assessed with a summative CBA including several clinical cases. This format of exam has remained unchanged since it was introduced in 2009. The clinical cases can been seen as a series of key features [11, 12], and are designed to simulate the management of real patients, with questions on history, physical exam, differential diagnosis and management. Clinical information is progressively delivered to the students in a sequential order, which has implications for the order in which questions need to be answered. Students can review prior information in each clinical case, but cannot modify their previous answers. This progressive delivery applies to all the items of the exam, whatever the format of the item.

### Measures

During the study period, paediatric CBAs used 212 Long-menu questions, 201 Pick N, 140 Type A, 3 numeric questions, and 2 matrices. The last two categories were not included in the study because they were not commonly used. Type A questions require the examinee to pick a single, best answer from a list of five options. In Pick N questions, examinees Pick N correct answers from a much longer list of options, usually 15 or more.

For a given item, the level of difficulty was defined as the average capacity of the students to find the correct answer, i.e. the percentage of correct answer. The power of discrimination was defined by the point biserial correlation: it evaluates the ability of the item to differentiate among students on the basis of how well they perform during the exam. In other words, it is an estimator of the degree to which a single item measures the same thing as all the other items of the exam.

### Analysis

Unless specified, data are summarized by the mean ± standard deviation. Multiple-way analyses of variance were performed to compare the formats, taking into account other factors: the year of examination, and the session depending on whether the exam was held in the middle of the clinical rotations, or close to the end of the rotations. Only the main effects were tested. One analysis of variance was made for the difficulty, and one for the discrimination. All analyses were run on R version 3.2.2 (The R Foundation for Statistical Computing) and TIBCO Spotfire S + ® 8.1 for Windows (TIBCO Software Inc).

## Results

Overall, 13 exam sessions took place during the study period, with a total of 855 examinees and 558 items. There were an average number of 66 (±15) examinees, and 44 (±4) questions per exams. The average performance score of the students was 79.7 % (±4.1 %), and the average Cronbach alpha was 0.71 (±0.05). The analysis of variance showed a significant format effect regarding both the level of difficulty (*p*-value = 0.0045) and the power of discrimination (*p*-value < 0.0001). The main results are displayed in Table 1. Overall, Long-menu question were easier than Type A questions and had no significant difference in difficulty level when compared with Pick N questions. Long-menu questions were however more discriminative than both Type A and Pick N questions.

**Table 1:** Difficulty and power of discrimination

Type of Item	n	Difficulty	Difference with Long-menu (95 % confidence interval)	Discrimination	Difference with Long-menu (95 % confidence interval)
Type A	140	75.69 ± 19.85	-5.24^a^ (-9.37 -1.11)	0.222 ± 0.236	-0.074^a^ (-0.136 -0.013)
Pick N	201	78.72 ± 13.88	-2.96 (-6.65 + 0.72)	0.196 ± 0.214	-0.104^a^ (-0.159 -0.049)
Long-menu	212	81.57 ± 16.84		0.304 ± 0.284	
All	553	79.04 ± 16.82		0.244 ± 0.253	.

^a^significant difference at 5 % level

Regarding difficulty, both the year of examination and the session effect were significant (*p* = 0.0066, and *p* = 0.0116 respectively). The remaining three groups of students of one given year of examination (second session) tended to perform better than the two groups of the first session (+3.35; 95 % confidence interval +0.46 to +6.25).

## Discussion

Long-menu questions were more discriminating than Type A and Pick N questions in this retrospective study, without having a higher level of difficulty. In other words, they were more reliable in identifying students with higher grades than other types of questions. When students face a Long-menu question, the answer box is initially empty, as for open or free text questions. The problem-solving mechanism assessed with this format may therefore more closely resemble the management of a real patient in daily practice. The other two formats provide a list of options, from which examinees can select the best answer(s). This absence of cueing effect could explain the higher discriminative power of this type of question. Moreover, this better discrimination is not at the expense of an increased difficulty. This is particularly important for our paediatric CBA, which aims at ascertaining that students have reached a required level of knowledge and skills: there is no selection process at this point of the medical program.

Although creating Long-menu questions may appear complex, their development and implementation is reasonable. In order for options to appear after the examinees start entering their responses, a tailored list of possible answers, such as a list of laboratory exams or diagnoses needs to be created. These possible answers need to be customized according to the specific key element tested in the question. Reviewing this list probably is the most time-consuming part, in an attempt to provide all possible reasonable options, without oversimplifying the responses.

Moreover, scoring these questions is easy and straightforward, since it can be done automatically by the analysis and grading software program. Although concern was raised initially due to synonyms that exam authors might have overlooked, this problem has turned out to be overestimated: in our institution students very rarely leave comments explaining the would have liked to use a word or term not recognized by the CBA program. We provide CBA exercise sessions for the students prior to the first summative assessment. They quickly get used to the logic induced by the Long-menu format, and immediately learn to try other synonyms if the answer they want does not provide the adequate answer options from the program. One restriction of Long-menu questions is the need for correct spelling, though only three consecutive correct letters are in fact needed: this could be a potential barrier for students who are not native French speakers.

The retrospective observational design is a major limitation of this study: since different questions were used with different formats, the contents were likely to have had an impact of both difficulty and discrimination. A prospective study comparing the formats with the same question contents is needed to confirm our findings. Other limitations of this study include the fact that the data were collected by a single institution, and that this format was only tested at a single level (early clinical years of the medical curriculum). Also the statistics collected for the analyses were based on exams with a relatively modest number of students, and bias resulting from the association between the question formats and the targeted knowledge or skill may not be excluded. However, the study includes seven cohorts of medical students, with little change in the medical training program, in particular regarding the students’ learning objectives. Furthermore, the team in charge of developing the exam did not change during the study period.

## Conclusion

Long-menu questions seem to have a higher discriminatory power than other type of MCQs with no cost in terms of level of difficulty, but to confirm our findings, more evidence should be brought by other studies with a more specific design. As a mix of different formats is likely to increase the validity of an exam [6, 13], the introduction of Long-menu questions could help assess clinical reasoning in a more realistic approach to patient management than other item formats inherited from paper based examination.

### Ethics

This study was not reviewed by an ethics committee. Following a 2009 decision of the Ethics Committee of Geneva and the Teaching Committee Office of the faculty of medicine of Geneva, research projects in medical education dealing with existing anonymous data, and designed to evaluate the quality of the pre-grade or post-grade educational programs, are exempted from formal decision approval by the Ethics Committee. The retrospective analysis exclusively dealt with items used during exams (format, and psychometric characteristics). No individual data regarding any patients/participants/examinees were available.

## Abbreviations

CBA: Computer-based assessment
MCQ: Multiple choice question
